# Prophylactic Effect of *Lactobacillus plantarum* YS4 on Oxazolone-Induced Colitis in BALB/c Mice

**DOI:** 10.1155/2020/9048971

**Published:** 2020-08-12

**Authors:** Ruokun Yi, Fang Tan, Huayi Suo, Wenfeng Li, Xianrong Zhou, Jianfei Mu, Peng Xie, Xin Zhao

**Affiliations:** ^1^Chongqing Collaborative Innovation Center for Functional Food, Chongqing University of Education, Chongqing 400067, China; ^2^Chongqing Engineering Research Center of Functional Food, Chongqing University of Education, Chongqing 400067, China; ^3^Chongqing Engineering Laboratory for Research and Development of Functional Food, Chongqing University of Education, Chongqing 400067, China; ^4^Department of Public Health, Our Lady of Fatima University, Valenzuela 838, Philippines; ^5^College of Food Science, Southwest University, Chongqing 400715, China; ^6^School of Life Science and Biotechnology, Yangtze Normal University, Chongqing 408100, China; ^7^Institute of Animal Science Chinese Academy of Agricultural Sciences, Beijing 100193, China

## Abstract

In the present research, the effects of *Lactobacillus plantarum* YS4 (LP-YS4) on colitis were tested in an oxazolone-induced mouse model. BALB/c mice were induced by oxazolone and then treated with LP-YS4. The serum levels of mice were analyzed using commercial kits and the protein and mRNA expression levels of mouse colon tissue were detected by Western blotting and qPCR assay, respectively. The results demonstrated that LP-YS4 significantly (*P* < 0.05) increased the colon length and ratio of colon weight/length in mice with colitis and attenuated the negative effects of colitis. The results also showed that treatment with LP-YS4 significantly reduced the serum concentrations of ET-1, SP, and IL-10 while significantly increasing those of SS, VIP, and IL-2 in colitis mice (*P* < 0.05). In addition, LP-YS4 significantly increased the activities of GSH and SOD while decreasing those of MPO and MDA in the colon tissue of colitis mice (*P* < 0.05). LP-YS4 also significantly upregulated the mRNA and protein expression of c-Kit, eNOS, nNOSe, and SCF in colitis mice while significantly downregulating the relative expression of iNOS. In summary, LP-YS4 could reduce the negative effects of colitis, and such effects were better than those of the common probiotic *Lactobacillus bulgaricus*.

## 1. Introduction

Ulcerative colitis is a type of inflammatory bowel disease that can potentially lead to cancer. The age of onset of colitis is typically 20–50 years old, and it can seriously threaten the quality of life of patients [1]. The immunopathogenesis and immunosuppressive treatment of colitis are currently the research topics of significant interest. The research goals are to diagnose and treat colitis in order to prevent exacerbation of the disease [2]. The drugs used to treat colitis in the clinic often have adverse effects after their long-term application. Another crucial area of colitis research is focused on the discovery of functional foods that can prevent colitis without side effects [3]. Natural plants including *Aegle marmelos* Linn. also have the intervention effect on colitis [4]. A recent study has shown that the intestinal flora is closely related to colitis and that the intestinal flora participates in the mucosal immune response [5]. Bacteria are an important promoter of inflammatory bowel disease. The symptoms of colitis can be alleviated by regulating the intestinal flora, preventing floral imbalance, and increasing the number of probiotics [6].

Yak yoghurt is a natural fermented food that is rich in nutrients and is common in the minority areas of the Qinghai Tibet Plateau. Previous research has suggested that yak yoghurt exerts various physiological activities such as antioxidation, cholesterol reduction, and immunity enhancement [7]. The Qinghai Tibet Plateau has a specific climate and unique environment for the fermentation of yak yoghurt. Additionally, the availability of yak milk and special Tibetan fermentation utensils (e.g., certain fermentation microorganisms) can make the flavor and quality of yak yoghurt differ greatly from ordinary fermented milk [8]. A prior study on the intestinal physiological activity of lactic acid bacterial species in Yak yoghurt showed that the lactic acid-producing bacteria isolated from yak yoghurt had antioxidant and constipation preventing effects [9].

In this study, the potential effects of *Lactobacillus plantarum* YS4 (LP-YS4) on oxazolidone-induced colitis were investigated for the first time. The findings provide a possible foundation for further development of LP-YS4, especially its application in functional food or medicine.

## 2. Materials and Methods

### 2.1. Experiment Animal, Materials, and Reagents

Experimental strain: the strain was isolated from yak yoghurt in the Yushu area of Qinghai Province, China, by our team. It was named LP-YS4 and stored in China Center for Type Culture Collection (CCTCC, Wuhan, China, no. M2016750). The negative control strain LB was purchased from the CCTCC (no. AB 200048).

Fifty male BALB/c mice (6 weeks old) were purchased from the Experimental Animal Center of Chongqing Medical University (Certificate no. SYXK (Yu) 2017–0001).

Oxazolone was purchased from Sigma-Aldrich Co. LLC, USA; IL-2, IL-10, ET-1, SP, SS, and VIP serum cytokine kits were purchased from BioLegend Inc., USA; GSH, SOD, MPO, and MDA kits were purchased from Nanjing Jiancheng Bioengineering Institute, Nanjing, China; Trizol reagent, oligodT18, RNase, dNTP, MLV, primer, BCA protein quantitative kit, APS, TEMED, SDS-PAGE, PVDF membrane, first antibody, and second antibody were purchased from Thermo Fisher Scientific, Inc., USA.

### 2.2. Instruments and Equipment

iMark microplate reader was purchased from Bio-Rad, USA); StepOnePlus PCR instrument was purchased from Thermo Fisher Scientific, Inc., USA; Tanon 5200 chemiluminescence imager was purchased from Tanon Science and Technology Co., Ltd., China; SAS v9.1 statistical software package was purchased from SAS Institute Inc., USA.

### 2.3. Animal Grouping and Intervention

A total of 50 BALB/c male mice were assigned to five groups: model group, normal group, *Lactobacillus bulgaricus* (LB) treatment group, and high-dose (LP-YS4-H) and low-doses (LP-YS4-L) treatment groups, and there were 10 mice in each group. The mice in LB, LP-YS4-H, and LP-YS4-L groups were fed with 1.0 × 10^9^, 1.0 × 10^9^, and 1.0 × 10^8^ CFU/kg (0.2 mL living bacteria physiological saline) of each corresponding strain daily by oral gavage for 26 consecutive days, and normal and model groups were fed with 0.2 mL physiological saline. After 15 days of treatment, the abdomens of all mice were shaved with an area of 2 cm × 2 cm. The mouse abdomens in normal group were daub treated with 0.2 mL of 99% ethanol, with those in the remaining four groups being daub treated with 0.2 mL of oxazolidone solution (mass ratio = 3%, solvent = 99%). After treatment for 19 days, the mice were anesthetized. Then, the blunt head of a silicone tube was inserted into the intestinal tract from the anus of the mouse at a depth of 3.5 cm. The mice in normal group were administered with 0.15 mL of 50% ethanol solution, while those in the remaining four groups were administered with 0.15 mL of 1% oxazolidone solution (mass ratio = 50% solvent = 99% ethanol). Twenty seconds later, the catheters were removed, and the mice were lifted up by their tails for half a minute [10]. On the last day of treatment (day 26), all the mice were sacrificed by decapitation and their plasma samples and colon tissues were collected. The length and weight of the colon were documented.

### 2.4. Detection of Endothelin-1 (ET-1), Substance P (SP), Somatostatin (SS), and Vasoactive Intestinal Peptide (VIP) Concentrations in Serum Samples

The whole blood samples of mice were allowed to clot at room temperature for 1 h and then centrifuged at 4,500 rpm/min for 15 min. After collecting the serum samples, the concentrations of ET-1, SS, SP, and VIP were detected using commercial kits.

### 2.5. Determination of Interleukin-2 (IL-2) and Interleukin-10 (IL-10) Levels in Serum Samples

The mouse serum samples were prepared according to Section 2.4. Then, the serum levels of IL-2 and IL-10 cytokines were assessed using commercial kits.

### 2.6. Determination of Glutathione (GSH) Malondialdehyde (MDA), Myeloperoxidase (MPO), and Superoxide Dismutase (SOD) Activities in Colon Tissues

A mixture of 0.2 g colon tissue and 1.8 mL normal saline was prepared at 1 : 9 weight ratio. After homogenizing the mixture, the activities of GSH, MDA, MPO, and SOD in colon tissues were evaluated using commercial kits.

### 2.7. Pathological Observation of H&E Staining

The lesion site (1.5 × 1.5 × 0.5 cm) of colon was cut with a scalpel. The trimmed tissue and corresponding label were placed in 10% neutral formalin solution for 24 h. The colon tissue was dehydrated, embedded, sliced, dewaxed, stained, and then dehydrated, transparent, and sealed. Finally, the pathological state of colon tissue was observed under a microscope (BX43, Olympus, Tokyo, Japan).

### 2.8. qPCR Assay

Total RNA was isolated using RNAzol and then diluted to the final concentration of 1 *μ*g/*μ*L. For cNA synthesis, 5 *μ*L of the diluted RNA extract was taken, and cDNA was prepared using a reverse transcriptase kit. Then, the cDNA template (2 *μ*L) was added into 1 *μ*L of SYBR Green PCR master mix and 1 *μ*L of forward primer and reverse primer each (Table 1). qPCR amplification was carried out for 40 cycles under the reaction conditions of 95^o^C, 60 s: 95°C, 15 s; 55°C, 30 s; and 72°C, 35 s, followed by 1 cycle under the reaction conditions of 95°C, 30 s, and 55°C, 35 s. GAPDH was used as internal control for this determination, and the relative mRNA expression was measured according to the following equation: 2^−ΔCT^ = ΔCT_(detection gene)_ - ΔCT_(GAPDH)_ [11].

### 2.9. Western Blotting

100 mg of tissue samples was mixed with 10 *μ*L of PMSF and 1 mL of RIPA and then homogenized at 12000 r/min, 4^o^C for 5 min. Protein quantification was conducted using the BCA protein quantitative kit, and the protein samples were diluted to 50 *μ*g/mL. Then, the diluted protein and sample buffer were mixed at 4 : 1, heated at 100^o^C for 5 min, and ice-bathed for 5 min. Subsequently, acrylamide, starting buffer, resolving buffer, TEMED, 10% APS, and differentiated water were mixed in specific proportions, in order to prepare SDS-PAGE separation glue and concentration glue. The prestained samples and Protein Ladder were placed into the sample hole of the rubber sheet, respectively, and then the protein-containing SDS-PAGE glue was subjected to vertical gel electrophoresis for 50 min. After activation with methanol for 1 min, the PVDF were blocked with 5% skimmed milk in 1 × TBST solution for 1 h. Then, the blocked PVDF membranes were rinsed with 1 × TBST, followed by incubation with the primary antibody, at 25°C for 2 h. After washing with 1 × TBST for 5 times, the secondary antibody was incubated at 25°C for 1 h. Lastly, the protein bands were visualized by SuperSignal West Pico PLUS Chemiluminescent Substrate, and the images were captured using a chemiluminescence imager [12].

### 2.10. Statistical Analysis

The average value of three experimental results was determined, and the statistical software SAS 9.1 was used to analyze whether there was a significant difference between each group at the level of *P* < 0.05. Multiple comparisons were conducted using one-way ANOVA followed by Tukey's test.

## 3. Results

### 3.1. Effect of LP-YS4 on Colon Parameters

**Figure 1:**
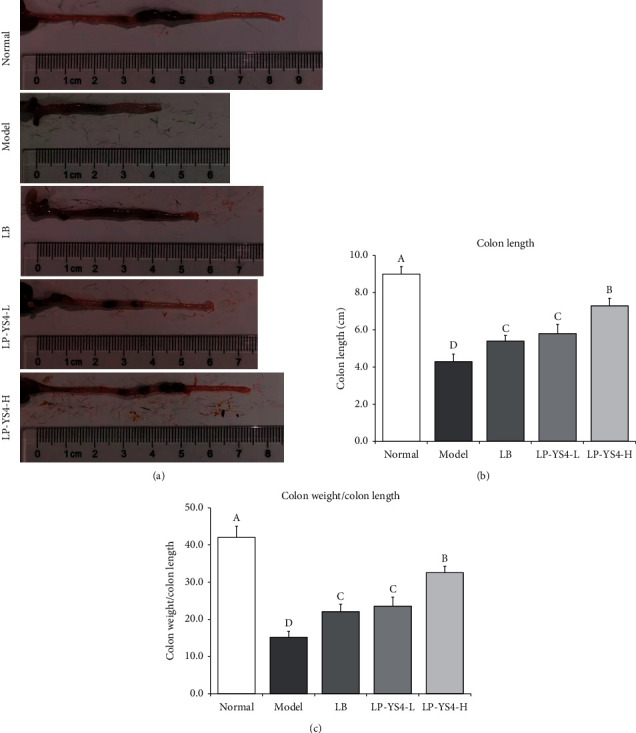
(a-c) Colon length and colon weight/colon length of each group of mice. Data are presented as the mean ± standard deviation. (A–D) Different letters mean values are significantly different (*P* < 0.05) according to Tukey's honestly significantly different test. LP-YS4-L: mice treated with a low concentration of *Lactobacillus plantarum* YS4 (1.0 × 10^8^ CFU/kg); LP-YS4-H: mice treated with a high concentration of *Lactobacillus plantarum* YS4 (1.0 × 10^9^ CFU/kg); and LB : mice treated with *Lactobacillus bulgaricus* (1.0 × 10^9^ CFU/kg).

The experimental results demonstrated that the length of mouse colon was the longest in normal group (9.0 ± 0.4 cm) while being the shortest in the colitis model group (4.3 ± 0.4 cm). Similarly, the ratio of colon weight/length was the highest in normal group (42.1 ± 3.0) while being the lowest in the colitis model group (15.2 ± 1.6, Figure 1). It was found that LP-YS4 could significantly (*P* < 0.05) attenuate the decline in colon length and weight/length ratio induced by colitis (5.8 ± 0.5 cm and 23.6 ± 2.4 for LP-YS4-L group; 7.3 ± 0.4 cm and 32.6 ± 1.7 for LP-YS4-H group), and these effects were better than those of LB (5.4 ± 0.3 cm and 22.1 ± 2.0).

### 3.2. Effect of LP-YS4 on the Serum Contents of ET-1, SP, SS, and VIP in Mice

**Figure 2:**
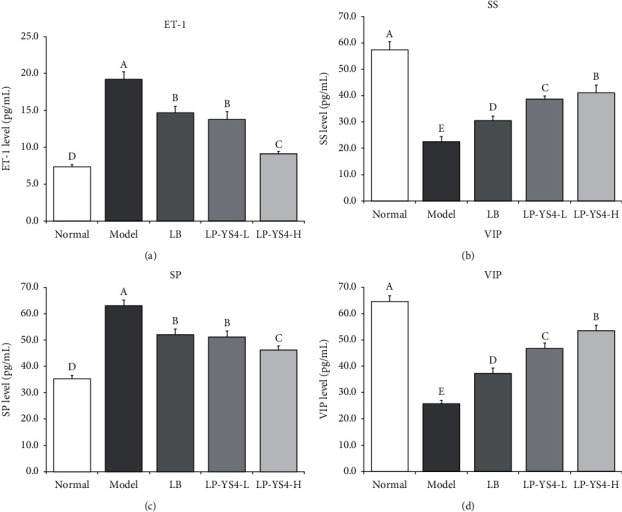
(a) ET-1, (b) SS, (c) SP, and (d) VIP serum levels of each group of mice. Data are presented as the mean ± standard deviation. (A–D) Different letters mean values are significantly different (*P* < 0.05) according to Tukey's honestly significantly different test. LP-YS4-L: mice treated with a low concentration of *Lactobacillus plantarum* YS4 (1.0 × 10^8^ CFU/kg); LP-YS4-H: mice treated with a high concentration of *Lactobacillus plantarum* YS4 (1.0 × 10^9^ CFU/kg); and LB : mice treated with *Lactobacillus bulgaricus* (1.0 × 10^9^ CFU/kg).

It can be seen from Figure 2 that the serum concentrations of SS (57.36 ± 3.11 pg/mL) and VIP (64.57 ± 2.20 pg/mL) in normal mice were increased compared to those in the remaining four groups. In contrast, the level of ET-1 (7.35 ± 0.28 pg/mL) and SP (35.27 ± 1.28 pg/mL) was decreased in normal mice compared to that of the remaining four groups. The colitis model mice exhibited the opposite results, in which the highest concentrations were observed for ET-1 (19.21 ± 1.02 pg/mL) and SP (63.02 ± 2.19 pg/mL), and the lowest concentrations were found for SS (22.47 ± 2.01 pg/mL) and VIP (25.79 ± 1.23 pg/mL). Interestingly, LP-YS4 could significantly (*P* < 0.05) improve the serum concentrations of SS and VIP in colitis mice and markedly decrease those of ET-1 and SP. More importantly, the effects were better after treatment with LP-YS4-H (41.05 ± 2.92, 53.52 ± 2.09, 9.12 ± 0.33, and 46.25 ± 1.51 pg/mL), and the effects of LP-YS4 were better than those of LB (30.52 ± 1.69, 37.29 ± 1.97, 14.68 ± 0.89, and 52.06 ±2.11 pg/mL).

### 3.3. Effect of LP-YS4 on the Serum Concentrations of IL-2 and IL-10 in Mice

**Figure 3:**
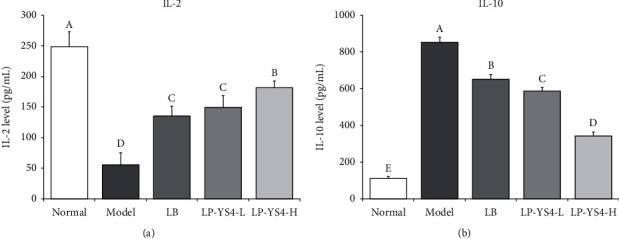
(a) IL-2 and (b) IL-10 serum levels of each group of mice. Data are presented as the mean ± standard deviation. (A–E) Different letters mean values are significantly different (*P* < 0.05) according to Tukey's honestly significantly different test. LP-YS4-L: mice treated with a low concentration of *Lactobacillus plantarum* YS4 (1.0 × 10^8^ CFU/kg); LP-YS4-H: mice treated with a high concentration of *Lactobacillus plantarum* YS4 (1.0 × 10^9^ CFU/kg); and LB : mice treated with *Lactobacillus bulgaricus* (1.0 × 10^9^ CFU/kg).

It can be seen from Figure 3 that the serum content of IL-2 (55.49 ± 19.75 pg/mL) and IL-10 (852.69 ± 28.39 pg/mL) in colitis model mice was significantly reduced and raised compared to the remaining four groups (*P* < 0.05). Following the treatment with LP-YS4, an increase in IL-2 and a decrease in IL-10 cytokine level were observed. Notably, the serum levels of IL-2 were markedly higher in LP-YS4-H- (55.49 ± 19.75 pg/mL) and LP-YS4-L-treated mice (149.55 ± 19.20 pg/mL) than in LB-treated mice 587.69 ± 19.85 pg/mL), and the serum levels of IL-10 were lower in LP-YS4-H- (181.68 ± 10.82 pg/mL) and LP-YS4-L-treated mice (342.58 ± 21.32 pg/mL) than in LB-treated mice (652.08 ± 25.37 pg/mL, *P* < 0.05).

### 3.4. Effect of LP-YS4 on the Activities of MPO, SOD, GSH, and MDA in Mouse Colon

**Figure 4:**
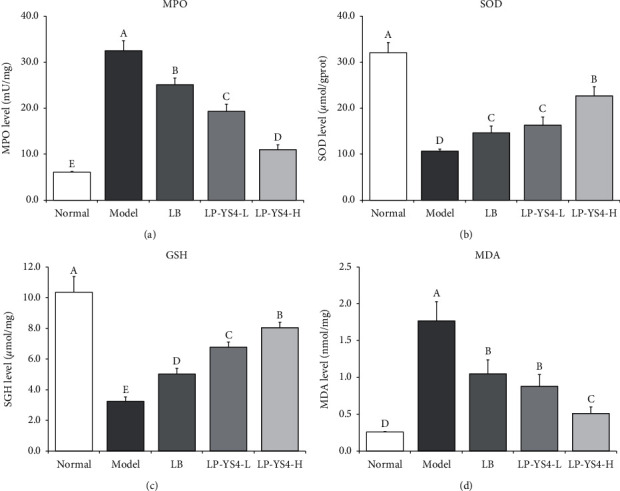
(a) MPO, (b) NO, (c) GSH, and (d) MDA colon tissue levels of each group of mice. Data are presented as the mean ± standard deviation. (A–E) Different letters mean values are significantly different (*P* < 0.05) according to Tukey's honestly significantly different test. LP-YS4-L: mice treated with a low concentration of *Lactobacillus plantarum* YS4 (1.0 × 10^8^ CFU/kg); LP-YS4-H: mice treated with a high concentration of *Lactobacillus plantarum* YS4 (1.0 × 10^9^ CFU/kg); and LB : mice treated with *Lactobacillus bulgaricus* (1.0 × 10^9^ CFU/kg).

As shown in Figure 4, the activities of GSH (10.36 ± 1.02 *μ*mol/mg) and SOD (32.06 ± 2.21 *μ*mol/gprot) in the colon tissue of the normal group were the highest while those of MPO (6.12 ± 0.13 mU/mg) and MDA (0.26 ± 0.04 nmol/mg) were the lowest among the five groups. After induced by oxazolidone, the levels of GSH (3.27 ± 0.29 *μ*mol/mg) and SOD (10.68 ± 0.41 *μ*mol/gprot) were significantly reduced (*P* < 0.05) while those of MPO (32.52 ± 2.17 mU/mg) and MDA (1.77 ± 0.26 nmol/mg) were remarkably increased (*P* < 0.05) in the model group mice. LP-YS4 could markedly attenuate the decline in GSH and SOD levels and prevented colitis-induced increase in MPO and MDA levels (*P* < 0.05). Such effect was stronger compared to LB-treated mice (5.03 ± 0.36 *μ*mol/mg, 14.66 ± 1.48 *μ*mol/gprot, 25.10 ± 1.48 mU/mg, and 1.05 ± 0.19 nmol/mg). More specifically, treatment with LP-YS4-H significantly increased the levels of (8.05 ± 0.35 *μ*mol/mg) and SOD (22.69 ± 1.98 *μ*mol/gprot) in colon tissue and markedly decreased those of MPO (10.98 ± 1.05 mU/mg) and MDA (0.51 ± 0.09 nmol/mg) when compared to colitis model group.

### 3.5. Pathological Observation

**Figure 5:**
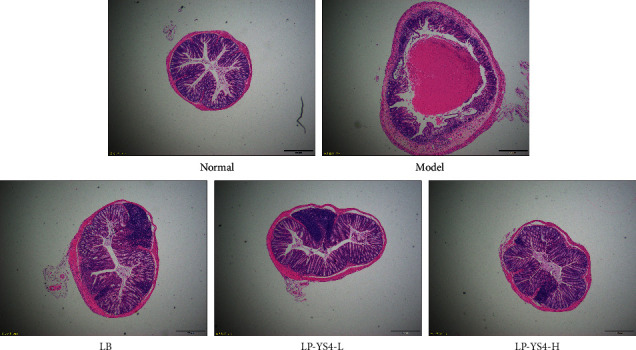
Observation of colon pathology in mice by H&E staining.

As shown in Figure 5, in the normal group, the epithelial cells of colon mucosa were intact, the inflammatory cells were normal without infiltration, and the goblet cells were arranged orderly, without congestion and edema. In the model group, the epithelial cells of colon tissue were obviously damaged, the intestinal wall was thickened, and edema, inflammatory cell infiltration, and goblet cells were reduced. After treatment with LB and LP-YS4, congestion, edema, and cell infiltration were alleviated, and the goblet cells were increased compared with the model group. Among them, LP-YS4-H had the most obvious effect on improving colon tissue, which indicated that LP-YS4 could reduce the colon injury caused by DSS, and the high efficiency effect was also enhanced with the increase of concentration.

### 3.6. Effect of LP-YS4 on mRNA and Protein Expression in Mouse Colon

**Figure 6:**
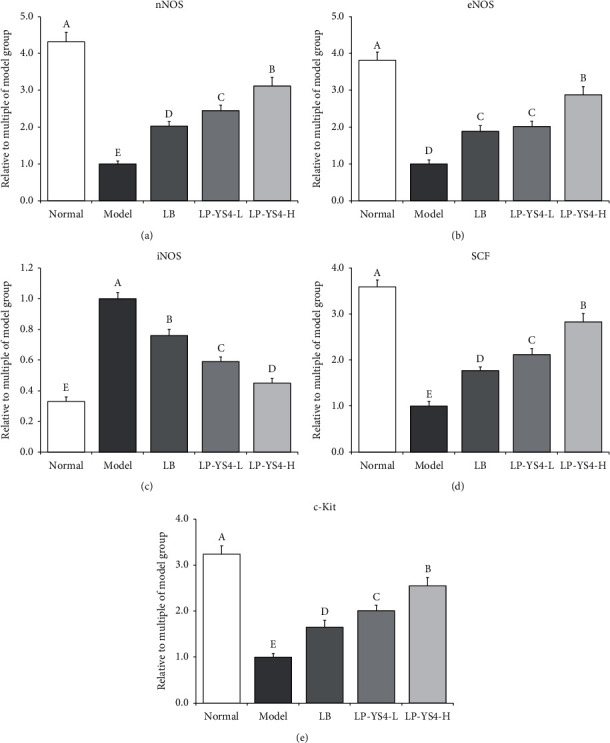
Effect of LP-YS4 on (a) nNOS, (b) eNOS, (c) iNOS, (d) c-Kit, and (e) SCF mRNA expression in mouse colon. Data are presented as the mean ± standard deviation. (A–E) Different letters mean values are significantly different (*P* < 0.05) according to Tukey's honestly significantly different test. LP-YS4-L: mice treated with a low concentration of *Lactobacillus plantarum* YS4 (1.0 × 10^8^ CFU/kg); LP-YS4-H: mice treated with a high concentration of *Lactobacillus plantarum* YS4 (1.0 × 10^9^ CFU/kg); and LB : mice treated with *Lactobacillus bulgaricus* (1.0 × 10^9^ CFU/kg).

**Figure 7:**
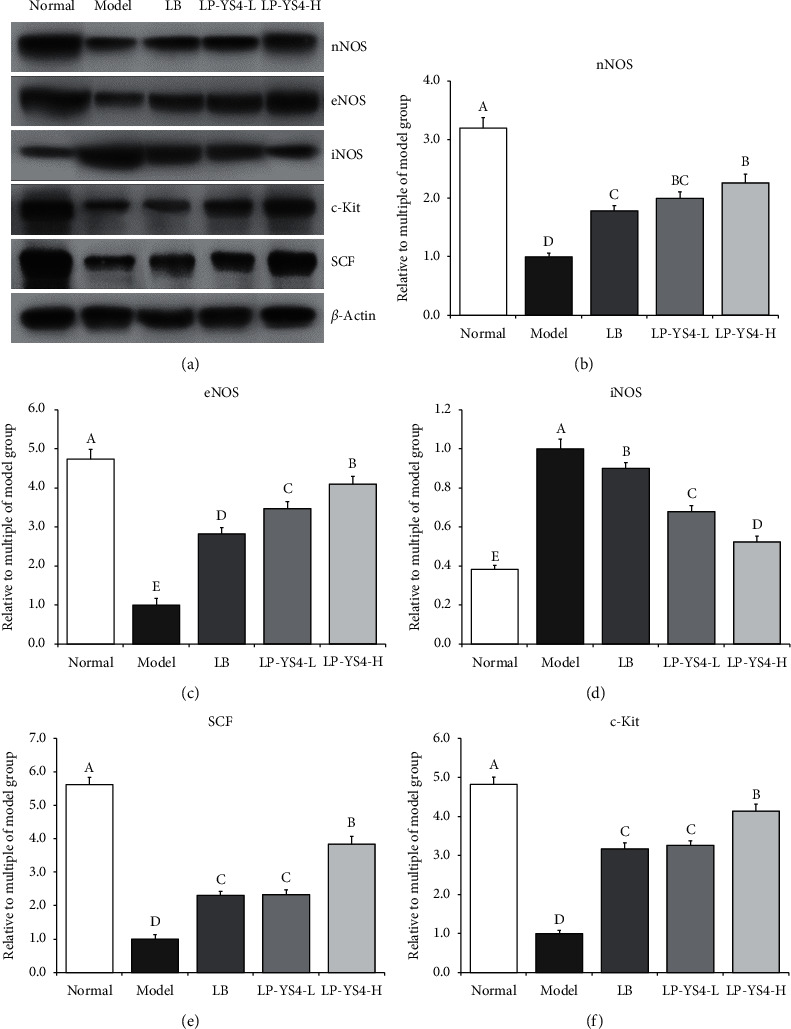
(a-f) Effect of LP-YS4 on nNOS, eNOS, iNOS, c-Kit, and SCF protein expression in mouse colon. Data are presented as the mean ± standard deviation. (A–E) Different letters mean values are significantly different (*P* < 0.05) according to Tukey's honestly significantly different test. LP-YS4-L: mice treated with a low concentration of *Lactobacillus plantarum* YS4 (1.0 × 10^8^ CFU/kg). LP-YS4-H: mice treated with a high concentration of *Lactobacillus plantarum* YS4 (1.0 × 10^9^ CFU/kg); and LB : mice treated with *Lactobacillus bulgaricus* (1.0 × 10^9^ CFU/kg).

As shown in Figures 6 and 7, colitis induction could lead to the upregulated mRNA and protein expression of iNOS in mouse colon but downregulated the relative expression of c-Kit, eNOS, nNOS, and SCF. Treatment with LP-YS4-H could increase the relative expression of nNOS, eNOS, c-Kit, and SCF and decrease that of iNOS in the colon tissues of colitis mice. Such effects were stronger than those of LP-YS4-L or LB treatment group.

## 4. Discussion

The ratio of colon weight/length is employed as a vital standard for assessing colitis *in vivo*. The colon length of colitis mice was shorter on average than that of control mice, and the ratio of colon weight/length was lower in colitis mice than in control mice [13]. However, it appeared that treatment with LP-YS4 could attenuate the decline in colon length and weight/length ratio induced by colitis.

A prior study has shown that vasoconstriction of the endothelin can lead to colonic mucosa erosion and ulceration, which in turn can exacerbate the progression of colitis [14]. SS, however, can reduce gastrointestinal inflammation by suppressing the production of gastric acid and other gastrointestinal fluids. Thus, a decrease in SS level can induce the secretion of gastrointestinal fluids, thus aggravating colitis [15]. Excessive accumulation of SP can induce colitis, but after antagonizing, it has been shown to relieve colitis *in vivo* [16]. VIP inhibits NO production by regulating the transcriptional activity of iNOS in the colon tissue, thus protecting the intestinal mucosa. Besides, VIP can also influence certain immune aspects of colitis [17]. In this study, LP-YS4 inhibited colitis by downregulating the levels of ET and SP and upregulating those of SS and VIP.

IL-2 is an effector cytokine produced by Th2 cells, which has been closely associated with colitis. Th2 cells regulate the inflammatory response that causes colitis, and IL-2 is involved in the suppression of inflammatory process and severity reduction of colitis by influencing Th2 cells [18]. IL-10 is another cytokine produced by Treg cells with immunoinhibitory effects, which plays a significant role in the development of colitis [19]. Inflammatory bowel disease (IBD) is a chronic refractory intestinal inflammatory disease, mainly including ulcerative colitis (UC) and Crohn's disease (CD). Although the etiology of IBD is still unclear, the imbalance between Th1 and Th2 has been recognized as the main cause of mucosal damage in IBD. Under the action of differentiation factor IL-10, regulatory T cells derived from intestinal mucosa associated lymphoid tissue can correct Th1/Th2 deviation by secreting high level of IL-10 and medium level of TGF-*β*, so as to achieve the purpose of treating inflammatory bowel disease to a certain extent [20]. It was observed that LP-YS4 could increase the level of IL-2, thus regulating immunity and alleviating colitis. And LP-YS4 could inhibit the colitis and reduce the secretion of IL-10.

The aggregation of neutrophils began to decline after intestinal inflammation, and a large amount of them entered into the circulation and migrated to tissues. At the same time, free radicals, such as reactive oxygen species and reactive nitrogen species, gathered in large quantities, which in turn led to damage and toxicity of colon tissue, and further aggravated colitis [21]. After colitis, the levels of SOD and GSH were reduced in colon tissue, while those of MDA and MPO were elevated. Our findings also indicated that colitis could lead to decrease in GSH and SOD levels as well as increase in MDA and MPO levels [10, 22]. In addition, LP-YS4 attenuated colitis by inhibiting the transcriptional responses to oxidative stress.

NOS can be divided into nNOS, eNOS, and iNOS. It has been reported that NO produced by eNOS plays a key role in response to colonic tissue damage, and excessive NO generated by iNOS promotes colitis damage [23]. eNOS controls the production of NO to keep the colonic tissue in a normal state, which plays an important role in reducing colitis-induced colonic injury. The presence of excessive NO aggravates colon damage [24]. nNOS can also control the level of NO in tissue and protect the tissue from being damaged by excessive NO [25]. In this study, LP-YS4 upregulated the expression of eNOS and nNOS in the colon and downregulated that of iNOS, thereby attenuating colitis.

Ulcerative colitis not only shows hematochezia and diarrhea, but also presents colonic motility disorders. It has been proved that interstitial cells of Cajal (ICC) are related to colonic motility dysfunction and directly participate in the progression of colitis. As a specific marker of gastrointestinal ICC, c-Kit is a transmembrane glycoprotein specifically expressed on ICC cell membrane. c-Kit gene, located on chromosome 4q12-13, belongs to proto-oncogene and its product is tyrosine kinase type III. As a receptor of SCF, c-Kit can regulate the proliferation and differentiation of hematopoietic stem cells through a series of signaling pathways [26, 27]. SCF exerts a direct effect on inflammatory bowel disease by regulating the function and number of ICC. SCF can interact with its ligand c-Kit, and the dysregulation of SCF/kit signaling pathway may decrease the proliferation and differentiation of ICC, thus exacerbating colitis [24, 27]. The abnormal expression of SCF/c-Kit signaling pathway can also change the physiological function of ICC, weaken gastrointestinal motility, and aggravate intestinal dysfunction [28]. In the present study, LP-YS4 could inhibit colitis by regulating the expression levels of SCF and c-Kit.

ICC autophagy regulation has become a new target for the treatment of intestinal motility disorder in ulcerative colitis [29]. Because the drug treatment of colitis often has side effects and once stopped, it is easy to relapse. Therefore, the use of natural harmless substances through the regulation of ICC prevention and treatment of colitis can maintain long-term health [30]. A study has shown that *Aurantii Fructus Immaturus* and *Atractylodis Macrocephalae Rhizoma* can inhibit the autophagy of Cajal stromal cells induced by glutamate, which may play an inhibitory role in colitis [31]. Meanwhile, there is also a study showing that lactic acid bacteria can regulate ICC, thus regulating intestinal function and protecting the intestine [32]. This study also confirmed that the LP-YS4 can regulate the SCF/c-Kit signaling pathway, and the SCF/c-Kit signaling pathway is an important ICC regulatory pathway [33]. Therefore, the LP-YS4 may also inhibit the colon by regulating ICC.

## 5. Conclusion

In this study, oxazolone was used to induce colitis in BALB/c mice, and the inhibitory effects of LP-YS4 on colitis were detected. Through the observation of colon tissues and serum samples of mice, it was found that LP-YS4 treatment could alleviate colitis by restoring the levels of inflammatory indicators closer to those measured in healthy control mice. This work suggests that LP-YS4 is superior-quality lactic acid bacteria with a potential role in colitis treatment and provides a foundation for further research and development.

## Figures and Tables

**Table 1:** Sequences of primers used in this study.

Gene name	Sequence
iNOS	Forward: 5′-AGAGAGATCGGGTTCACA-3′
	Reverse: 5′-CACAGAACTGAGGGTACA-3′
nNOS	Forward: 5′-TCGTCCAACTTCTGGGCTCTT-3′
	Reverse: 5′-CCTTCTCTTCCTCCCCTCTCTTC-3′
eNOS	Forward: 5′-TCAGCCATCACAGTGTTCCC-3′
	Reverse: 5′-ATAGCCCGCATAGCGTATCAG-3′
c-Kit	Forward: 5′-CATAGCCCAGGTAAAGCACAAT-3′
	Reverse: 5′-GAACACTCCAGAATCGTCAACTC-3′
SCF	Forward: 5′-TCAGGGACTACGCTGCGAAAG-3′
	Reverse: 5′-AAGAGCTGGCAGACCGACTCA-3′
GAPDH	Forward: 5′-TGCACCACCAACTGCTTAG-3′
	Reverse: 5′-GATGCAGGGATGATGTTC-3′

## Data Availability

No data were used to support this study.

## Abbreviations

LP-YS4: *Lactobacillus plantarum* YS4
LB: *Lactobacillus bulgaricus*
qPCR: Quantitative polymerase chain reaction
H&E: Hematoxylin-eosin
ET-1: Endothelin-1
SP: Substance P
SS: Somatostatin
VIP: Vasoactive intestinal peptide
IL-2: Interleukin-2
IL-10: Interleukin-10
MPO: Myeloperoxidase
SOD: Superoxide dismutase
GSH: Glutathione
MDA: Malondialdehyde
eNOS: Endothelial nitric oxide synthase
nNOS: Neuronal nitric oxide synthase
iNOS: Inducible nitric oxide synthase
SCF: Stem cell factor.
